# The Implementation of Mass Spectrometry-Based Proteomics Workflows in Clinical Routines of Acute Myeloid Leukemia: Applicability and Perspectives

**DOI:** 10.3390/ijms21186830

**Published:** 2020-09-17

**Authors:** Maria Hernandez-Valladares, Øystein Bruserud, Frode Selheim

**Affiliations:** 1 Department of Clinical Science, University of Bergen, 5021 Bergen, Norway; 2 The Proteomics Facility of the University of Bergen (PROBE), Department of Biomedicine, University of Bergen, 5009 Bergen, Norway; 3 The Department of Biomedicine, University of Bergen, 5009 Bergen, Norway

**Keywords:** acute myeloid leukemia, clinical proteomics, diagnosis, prognosis, treatment, biomarker, bioinformatics pipeline, laboratory robots

## Abstract

With the current reproducibility of proteome preparation workflows along with the speed and sensitivity of the mass spectrometers, the transition of the mass spectrometry (MS)-based proteomics technology from biomarker discovery to clinical implementation is under appraisal in the biomedicine community. Therefore, this technology might be implemented soon to detect well-known biomarkers in cancers and other diseases. Acute myeloid leukemia (AML) is an aggressive heterogeneous malignancy that requires intensive treatment to cure the patient. Leukemia relapse is still a major challenge even for patients who have favorable genetic abnormalities. MS-based proteomics could be of great help to both describe the proteome changes of individual patients and identify biomarkers that might encourage specific treatments or clinical strategies. Herein, we will review the advances and availability of the MS-based proteomics strategies that could already be used in clinical proteomics. However, the heterogeneity of complex diseases as AML requires consensus to recognize AML biomarkers and to establish MS-based workflows that allow their unbiased identification and quantification. Although our literature review appears promising towards the utilization of MS-based proteomics in clinical AML in a near future, major efforts are required to validate AML biomarkers and agree on clinically approved workflows.

## 1. Introduction

In the last two decades, liquid chromatography-tandem mass spectrometry (LC-MS/MS) technologies have provided a large number of candidate biomarkers in many diseases. However, the translation of these candidate biomarkers into clinical use has been limited. An early study on plasma proteomic biomarkers for cardiac transplantation highlighted the need for a robust analytical methodology to transition from biomarker discovery to clinical implementation [1]. A three-stage computational pipeline that provided a systematic process from discovery to validation and to clinical implementation of plasma protein biomarkers was described. That comprised of an initial untargeted exploration of the plasma proteome resulting in a list of potential biomarkers, followed by a quality assessment of candidate biomarkers and the combination of some of them into a classifier score with clinical utility. Both internal validation -by multiple reaction monitoring mass-spectrometry (MRM-MS), enzyme-linked immune-sorbent assay (ELISA) and immunonephelometric assay (INA) as in this study- and validation of the classifier score on large external cohorts were required before clinical implementation. Therefore, external validation represents a major challenge for most of the clinically orientated biomarker discovery studies due to the lack of external cohorts or research collaborations for their use. Alternatively, the LC-MS/MS-quantified expression of candidate biomarkers might be compared to the protein expression levels obtained from tumors or cell lines samples using reverse-phase protein arrays (RPPAs) with extensively validated antibodies at The Cancer Proteome Atlas (TCPA) [2]. 

Clinicians may use one or more approaches to diagnose most cancers. These include a physical exam, laboratory tests (mainly of blood and urine samples), imaging strategies, and biopsy which will be processed for histological analysis. Molecular tests in clinical oncology, also termed precision medicine, are used to confirm the disease and classify the tumor. They can predict prognosis and for certain malignancies guide clinicians to most efficient therapies and personalized selection of drugs. Broad molecular profiling can also be useful for the prognostication of patients with metastatic solid tumors [3]. Currently, the most used molecular profiling techniques which examine nucleic acids are polymerase chain reaction (PCR), reverse transcription PCR, in situ hybridization (ISH), and next-generation sequencing (NGS). To determine the levels of protein expression in tissue samples, e.g., tumor-specific antigens or tumor cell proliferation markers, ELISA and immunohistochemistry (IHC) with highly specific antibodies are widely utilized in the clinic [3,4]. Most of these techniques measure one or a few proteins.

Omics technologies have revolutionized disease research in the last two decades. Proteomics aims to identify and quantify the protein content of any biological sample as well as the multiple protein modifications and protein interactions. Thus, MS technology is capable of measuring many proteins at once and characterize whole pathways or whole protein assemblies that would lead to much better diagnoses, disease classification, and treatment options [5]. Common sample types in cancer clinical research include tumors, plasma, urine, and other body fluids. Future clinical samples might include exosomes which are nano-size membrane vesicles containing unique protein cargoes that reflect the tumor characteristics and microenvironment of potential diagnostic and prognostic use [6]. The standard bottom-up or shotgun proteomics strategy employs MS to analyze peptides obtained from protein digests and separated by chromatography to improve signal-to-noise, to increase proteome coverage, and to reduce interference between peptides in quantitative procedures. The continuous development of the MS technology dramatically improving the resolution, mass accuracy and speed of the equipment boosts the utilization of this technology in molecular medicine. The MS instruments determine the mass-to-charge ratio of each peptide in the sample and further fragments each of them to allow determination of the amino acid sequence by database searching in subsequent data analysis steps. Relative quantification is carried out with metabolic labeling with heavy amino acids, i.e., stable isotope labeling with amino acids in cell culture (SILAC), or chemical labeling with isobaric tags such as isobaric tag for relative and absolute quantitation (iTRAQ) or tandem mass tag (TMT). Peptide samples can be combined with heavy proteins or peptides for absolute quantitation of proteins in targeted proteomics workflows. Further processing of MS raw files by proteomics software provides the identification and quantification of thousands of peptides and proteins in the study sample.

**Figure 1:**
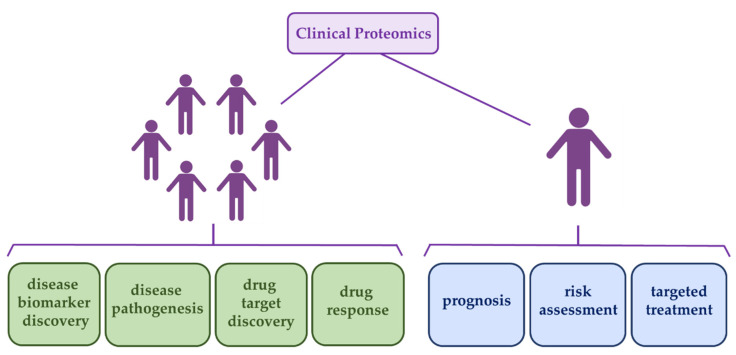
Most potential applications of clinical proteomics in human disease. Population-based applications are shown in green boxes and individual-based applications (also known as personalized medicine) are shown in blue boxes. The human figure was obtained from BioRender (https://biorender.com/).

Although MS-based proteomics has been mainly employed in discovery approaches in the past years, it is becoming more and more used as a validation approach of biomarker candidates and applied to routine diagnostics and prognostics in the clinic by examining predetermined peptides according to the targeted proteomics strategy. The targeted approach is highly suitable when medium-high sensitivity, high specificity, and easy multiplexing are required [7]. Other potential applications of MS-based proteomics in clinical proteomics are illustrated in Figure 1.

Acute myeloid leukemia (AML) is an aggressive hematological cancer [8,9]. Acute promyelocytic leukemia (APL) is a subset of patients with specific genetic abnormalities and receiving a specific treatment different from other AML patients [10,11]. In the present review, the term AML refers to the non-APL variants of AML. Patients with non-APL are highly heterogeneous regarding genetic abnormalities and prognosis, with a median age at the time of the first diagnosis of 65–70 years [12]. Despite the heterogeneity, AML patients are treated according to the same guidelines both with regard to intensive and potentially curative treatment, including allogeneic stem cell transplantation (ASCT) for the young and fit subset of patients, and less intensive leukemia-stabilizing treatment for the elderly (i.e., above 70–74 years of age) or unfit patients [13,14,15]. Most patients receiving intensive anti-AML treatment achieve complete hematological remission, but a major cause of death is due to primary resistance and chemoresistant leukemia relapse either during or following the chemotherapy [13,14,16]. The overall long-term AML free survival even for young and fit AML patients are therefore only approximately 50% [12]. Thus, there is a need for better prognostic classification and therapeutic strategies for AML patients.

In the present manuscript, we will review recent advances, applicability, and implementation of the MS-based proteomics technology for clinical routines and focus on the application of MS-based approaches to provide disease biomarkers as well as the potential application of global and targeted proteomics on AML samples in the clinic. Despite the large capacity of the MS approach, we will address the areas that need to be optimized before adopting MS-based workflows into clinical cancer research and especially in AML.

## 2. AML Heterogeneity and Prognostic Evaluation

As the median age at the time of diagnosis is so old for AML patients, many unfit or elderly patients cannot receive the usual intensive treatment due to the unacceptable risk of treatment-related mortality [12]. For these patients leukemia-stabilizing treatment is possible, but the effect of such treatment varies between patients and many of them have a short survival of only a few months [17,18,19,20]. Furthermore, during the last decade, the biological heterogeneity with regard to leukemogenesis and chemosensitivity in AML has been extensively studied and many possible therapeutic strategies have been suggested based on experimental in vitro and in vivo studies [21,22,23]. However, even though four different approaches were recently approved for AML treatment, these strategies included more optimal use of well-known drugs or drugs that can be used only for subsets of patients [24,25,26,27,28,29,30,31,32].

### 2.1. Prognostic Evaluation as the Basis for Therapeutic Strategies

#### 2.1.1. Non-Relapse Mortality Due to Intensive Antileukemic Therapy

AML is a very aggressive disease that can be cured only by intensive antileukemic treatment, i.e., high-dose induction (seven days of nucleoside analog cytarabine and three days of an anthracycline antibiotic, daunorubicin or idarubicin) and consolidation conventional chemotherapy (either high-dose, 3 g/m^2^, monotherapy [33] or intermediate-dose, 1 g/m^2^, cytarabine in combination with one or two additional cytotoxic drugs) possibly combined with ASCT [12]. The more intensive treatment, i.e., the stronger the antileukemic effect, the higher is the risk of severe treatment-related morbidity and mortality. This risk of non-relapse mortality is higher for elderly and unfit patients; these patients may also have complicating diseases that will increase this risk. Comorbidity scores can be used both for patients receiving conventional chemotherapy and for patients treated with ASCT [34,35,36,37,38,39,40]. Several scores are now available, including the comorbidity index that is based on whether the patients have other complicating diseases that will increase the risk of severe treatment-related morbidity or mortality [41] and the European Group for Blood and Marrow Transplantation (EBMT) index that mainly evaluates the risk based on transplantation-immunological factors and disease stage/status [42].

#### 2.1.2. The Factors Currently Used for Evaluation of Relapse Risk in Patients with Newly Diagnosed AML

A careful prognostic evaluation at the time of the first diagnosis is needed for all AML patients together with the evaluation of comorbidity and risk of severe toxicity with regard to ASCT. The intention is then to clarify whether (i) the risk of severe toxicity when trying intensive and potentially curative intensive chemotherapy possibly combined with autologous stem cell transplantation (AuSCT) is acceptable/low; and (ii) conventional intensive therapy can be used or the risk of a chemoresistant relapse is so high that one should recommend the much more toxic but at the same time more effective ASCT as part of the treatment.

AML is a very heterogeneous disease and this heterogeneity has been demonstrated both by cytogenetic and molecular genetic analyses as well as gene expression, epigenetic, and possibly also proteomic and metabolic profiling [12,43,44,45,46,47,48]. The most important factors for evaluation of AML relapse risk in routine clinical practice are the cytogenetic and the molecular genetic abnormalities (these and other prognostic factors are gathered in Table 1). Based on the genetic abnormalities, patients can be classified into favorable, intermediate, and adverse prognosis and these groups will usually have a risk of chemoresistant relapse corresponding to 30–35%, 45–55%, and 80–85%, respectively [12]. The most intensive treatment with ASCT will often be justified for patients with intermediate/adverse prognosis and with low comorbidity and thereby an acceptable risk of severe toxicity after allotransplantation [12].

High levels of circulating leukemia cells at the time of diagnosis have been regarded as an adverse prognostic impact in human AML, but this impact is weak compared with the genetic abnormalities [49]. The same conclusions have been reached in more recent studies; it may have an impact on certain subsets of patients but this seems to be true mainly for extreme cell levels exceeding 50–100 × 10^9^/L [62,63,64].

The response to the antileukemic treatment also has a prognostic impact [65]. Firstly, the number of remaining AML cells detected by morphological bone marrow examination 14 days after the start of conventional therapy reflects the AML cell chemosensitivity, and >10% remaining leukemia cells is associated with an adverse prognosis [55]. Second, most patients reach complete hematological remission after the first induction chemotherapy cycle; the absence of remission at this time point is associated with an adverse prognosis [49]. Finally, remaining minimal residual disease (MRD) detected after the start of consolidation therapy is also associated with an adverse prognosis and this parameter seems to be useful especially for the further subclassification of patients with an intermediate risk based on genetic analyses alone [56,57].

Several studies of patients with various malignancies have shown that the acute phase reaction, i.e., systemic inflammatory responses, have an adverse prognostic impact in several malignancies, including certain hematological malignancies [66]. However, systemic inflammatory activity has not been used for prognostication in human AML. The only systemic effect of the disease that seems to have a prognostic impact is the altered metabolic regulation reflected in the plasma or serum levels of various metabolites. A large clinical study showed that the systemic levels of 14 glucose metabolites could be used to identify patients with an increased risk of later leukemia relapse [51]. Other studies also suggest that the AML cell metabolism and not only the systemic metabolic regulation differ between AML patients [48,67,68], but the possible prognostic impact of such differences needs further investigation.

Patients with AML secondary to previous chemotherapy or previous chronic myeloid diseases (i.e., myelodysplastic syndrome or chronic myeloproliferative neoplasia) also have an adverse prognosis, and this is true especially for younger adults [50]. Another adverse prognostic parameter is the identification of AML subclones at the time of diagnosis [52,53,54].

Finally, aging by itself may also represent an unfavorable risk factor, possibly due to general age-dependent alterations that are also transmitted to the AML cells [69].

#### 2.1.3. Prognostic Evaluation of Patients with Relapsed AML

An AML relapse is always more chemoresistant than the disease at the first time of diagnosis and ASCT will usually be the only realistic alternative for a cure. A short duration (i.e., less than 9–12 months) of the first remission is usually more chemoresistant and the possibility of reaching a second complete remission is usually lower than for patients with longer duration of the first remission [58,59]. This is also true for AML relapse after ASCT; the later the relapse the better chance of reaching a new complete remission with the possibility of a retransplantation [60,61].

#### 2.1.4. Prognostic Evaluation of Patients Receiving AML Stabilizing Treatment

Prognostic factors for patients receiving AML stabilizing treatment are not necessarily the same as for patients receiving conventional chemotherapy. This is illustrated by the use of disease-stabilizing treatment based on drug combinations including the histone deacetylase inhibitor plus all-trans retinoic acid together with low-toxicity chemotherapy. In one study several of the responders had AML relapse [70] and responsiveness to therapy was associated with a metabolic profile with differences in lipid and amino acid metabolites but not in glucose metabolism [71] as described for conventional intensive chemotherapy. 

### 2.2. Improved Prognostic Evaluation and More Extensive Molecular Characterization of Therapeutic Targets Are Needed in AML

Based on the brief description above it should be clear that a better pretreatment prognostic evaluation is needed for patients receiving intensive AML therapy. Genetic analyses are the most important strategy for the prognostic evaluation, but even patients with favorable genetic abnormalities have a relapse risk of 30–35% [12]. It would be an advantage to better characterize these patients before the first induction cycle so that the initial treatment could be intensified and the risk of later, more chemoresistant, relapse thereby could be avoided. The same is true for the large patient group of intermediate risk. Although the evaluation of MRD during consolidation therapy would probably be helpful, it does not solve all problems because a subset of patients might not have suitable genetic markers or flow cytometric molecular profiles for MRD evaluation. Moreover, even patients without detectable MRD still have a risk of relapse. Thus, additional proteomic profiling of these patients may then become helpful.

Several new possible therapeutic protein targets have been identified in human AML [72]. Reviewing all AML targets identified by LC-MS/MS is beyond the scope of this review. As the number of identified targets has notably increased in the last decade, proteomic and phosphoproteomic profiling may then become useful for a more detailed biological/molecular characterization of AML patients. Proteomic and phosphoproteomic studies may not only be useful for the identification of new therapeutic targets but they could also be used for the design of combining new therapeutic strategies in patients receiving potentially curative intensive therapy or for studying those patients who might only receive AML-stabilizing treatment. 

An important question to address is what kind of AML cells should be examined in future prognostication studies of AML patients. AML relapse is the most important cause of death for patients receiving conventional intensive chemotherapy and it is still one of the most important causes of death in allotransplant recipients. However, relapse is thought to be derived from AML stem cells [65], and investigating a small leukemic stem cell population will limit the number of proteins that can be studied by LC-MS/MS.

Regarding prognostication, several observations have suggested that studies of the whole hierarchically organized AML cell population can be relevant. First, early morphological disease control with remission induction (but remaining MRD) is an important prognostic parameter [13,14,73,74] and this is a characteristic of the total AML cell population. Second, a large number of clinical studies have shown that biological characteristics of the total AML cell population reflect fundamental cell population characteristics that have a prognostic impact and are essential for leukemogenesis/chemosensitivity [43,44,46,47,48]. Third, previous studies have shown that the AML stem cells can be detected in various subsets, usually CD34^+^CD38^−^ but also CD34^−^ and CD34^+^CD38^+^ cells [65,75,76]. However, stem cell characteristics are also reflected in the overall cell population and therefore they can be detected as prognostic parameters also when investigating such populations [76].

Taken together those considerations the proteome and modified proteome description of AML patients, characterized when using the total AML cell population, represent a relevant and necessary support to the standard genetic characterization of AML patients both at the time of the first diagnosis and at a time of relapse.

## 3. Recent Advances in LC-MS/MS-Based Proteomics and Phosphoproteomics for AML Blast Cells

We have previously reviewed AML discovery-based proteomics with the main focus on AML patient material, sample preparation, and identified markers [72]. These first studies were mostly performed with AML cell lines, or small patient cohorts, with low-performance polyacrylamide gel electrophoresis (1D-PAGE and 2D-PAGE)-based fractionation methodologies prior to matrix-assisted laser desorption/ionization-time of flight (MALDI-TOF) MS or LC-MS. The recent improvement in sample preparation in combination with the high sensitivity and scan speed of the modern mass spectrometers has allowed high-throughput quantification of thousands of AML disease-related proteins (including their phosphorylation status). We will here describe the late progress in protein profiling of primary human AML cells, including pre-analytical sample preparation, as well as LC-MS/MS-based proteomics and phosphoproteomics workflows from the most recently published studies (Table 2) and assess their utility in clinical proteomics of AML cells. Readers are referred to previous reviews for older studies [72,77,78,79].

**Figure 2:**
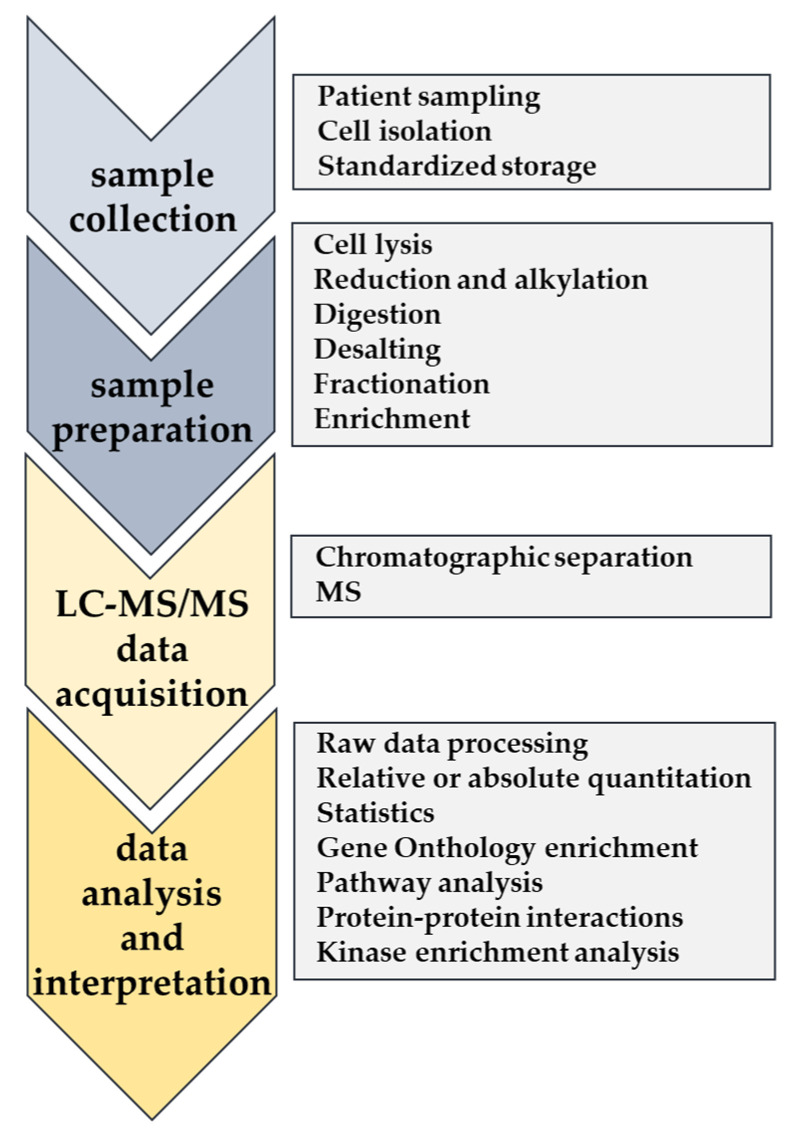
Workflow of standard mass spectrometry (MS)-based proteomics with a description of main tasks at each step.

Sample collection and preparation are crucial steps in proteomics workflows prior to LC-MS/MS analysis (Figure 2). During the past years, numerous different sample preparation methods have been used to prepare AML cells for LC-MS/MS analysis [72]. However, a consensus protocol for patient sample preparation that could be used in standard clinical routines has not been established yet. Standardized sample preparation protocols should therefore be developed considering the sample’s availability and the type of analysis (e.g., proteomics or phosphoproteomics) to carry out.

Thus, a Standard Operating Procedure (SOP) for MS-based clinical proteomics is warranted to achieve reproducible and efficient lysis and protein denaturation of the primary AML cells, reduction of the disulfide bonds before alkylation, and subsequent protease digestion of proteins into peptides.

Denaturants, e.g., urea, guanidinium hydrochloride and trifluoroethanol (TFE), which induce cell lysis, and detergents, e.g., sodium dodecyl sulfate (SDS), sodium deoxycholate (SDC) and 3-[(3-cholamidopropyl)dimethylammonio]-1-propanesulfonate (CHAPS), which disrupt cell membranes, are commonly used to extract proteins from cell samples. SDS is a superior agent also for solubilization and denaturation of proteins, but it will interfere with downstream enzymatic digestion, reversed-phase separations and MS, and must therefore be removed from the sample preparations [91]. Removal of contaminants and SDS can be accomplished with the filter-aided sample preparation (FASP) method, which enables proficient wash out of SDS (≥99.9%) with urea [92,93,94]. To further remove MS-incompatible and interfering chemicals, a clean-up method (also called desalting) of the peptide sample is usually performed before the LC-MS/MS data acquisition [91]. Moreover, the sample complexity of the peptide mixture can be reduced by fractionation and/or enrichment prior to the LC-MS/MS analysis. The most common fractionation protocols for complex peptide mixtures use chromatographic columns or self-packed microcolumns in stage-tips with e.g., ion exchange or hydrophilic interacting (HILIC) material [95]. The high-pH reversed-phase methodology has recently shown a superior fractionation when compared to those that employ ion exchangers [96].

Popular relative quantitative proteomics use SILAC [97] and the super-SILAC mix [98], TMT labeling [99], and label-free approaches [100]. SILAC involves selectively labeling of the proteomes to be compared either with a heavy or light normally occurring amino acid. Due to the difference in mass between the isotopes of the SILAC amino acid, differentially labeled peptides can be quantified by MS. The super-SILAC mix or spike-in SILAC standard is a mixture of multiple SILAC-labeled cell lines derived from a specific disease. The heavy labeled diagnostic peptides from the SILAC mixture will serve as an internal standard (IS) in the individual patient samples. Hence, a ratio is obtained between the “heavy” and “light” versions of the peptides for each sample, and since the amount of “heavy” peptide is added in a constant amount in all samples, a relative quantification between the different patient samples can be obtained. An AML-super SILAC mix made up of five cancer-derived AML cell lines (THP-1, NB4, MV4-11, Molm-13, and OCI AML) harboring different mutations, was found to have high proteome coverage compared to single cell lines, as well as high technical reproducibility. Moreover, this AML specific IS was able to quantify efficiently disease-related proteins in patient cells [101].

We adapted the AML-super SILAC mix approach to compare traditional and new sample preparation methods on AML patient cells: single vs. double digestion; in-solution (urea and guanidinium hydrochloride) vs. FASP; with and without peptide fractionation (i.e., unfractionated sequential elution of proteolytic digests, mixed-mode fractionation with polystyrene divinylbenzene reversed-phase sulfonate, SDB-RPS, and strong cation exchange, SCX, plugs). The FASP protocol combined with the mixed-mode fractionation produced the highest numbers of quantified proteins and peptides and had a lower percentage of missed cleavages [102,103].

The enrichment of phosphopeptides is performed with immobilized metal affinity chromatography (IMAC) [104], metal oxide affinity chromatography (MOAC) [105], and sequential elution from IMAC (SIMAC) [106]. We tested these three phosphopeptide enrichment approaches on the AML-derived peptide solutions prepared by FASP as described above [102,103]. We found FASP combined with the IMAC phospho-enrichment procedure to be the superior phosphoproteomic workflow. The reliable protocol for optimal quantitative proteomic and phosphoproteomic analysis on density gradient-separated AML patient cells is described step by step elsewhere [102,103]. We have recently managed to quantify as much as 6781 SILAC-labeled proteins and 12,309 class I phosphosites from only 20 µg and 600 µg (on average) as AML protein amounts, respectively, using blast cells extracted from the peripheral blood of patients at the time of first diagnosis [46]. Patients who relapse after intensive chemotherapy, compared to long-term (>5 years) leukemia-free survivors, were associated with a low abundance of V-ATPase proteins and a high abundance of RNA processing proteins, as well as with high casein kinase 2 (CSK2) and cyclin-dependent kinases (CDKs) activities. An additional strength of the super-SILAC mix approach is that one can directly correlate phosphosite regulation to protein expression. By using the AML specific IS, we found that 50% of the perturbed specific phosphosites were due to increased kinase-specific phosphorylation activity in AML cells from patients that would later relapse and not by protein expression changes of the matching protein [46]. Our super-SILAC mix approach has also been used successfully to study PI3K-Akt-mTOR signaling [68,85] as well as the prolonged self-renewal capacity of leukemic stem cells (LCSs) [86] (Table 2).

The LC-MS/MS-based super-SILAC mix technique in combination with the SCX fractionation and subsequent IMAC enrichment of phosphopeptides has previously been utilized on urea lysates from density-gradient isolated bone marrow aspirates by another research group [107]. By comparing the proteome and phosphoproteome from responding and non-responding AML patients to the FLT3 inhibitor quizartinib, they found several differentially regulated phosphorylated sites, including S630 located on B-cell lymphoma/leukemia 11A (BC11A) protein, upregulated in the non-responding patient group. As similarly demonstrated by our group recently, several specific phosphosites, like BCL11A S630, were significantly upregulated in the patients who relapse after intensive chemotherapy [46]. Thus, increased phosphorylation of BCL11A S630 seems to be associated with a poor outcome for patients with AML.

In another recent study, we compared proteomic and phosphoproteomic profiles for paired AML cell samples derived at the time of primary diagnosis and later, after completed intensive chemotherapy, at the time of the first relapse from the same patients [47]. Interestingly, the high CDK activity detected at diagnosis for patients who later relapsed was also observed in chemoresistant cells after relapse. In addition, high levels of proteins important for various mitochondrial functions, RNA processing, and DNA repair were detected in chemoresistant cells after relapse.

Recent evidence suggests that LSCs with mainly the CD34^+^CD38^−^ immunophenotype, a subpopulation of AML cells with self-renewal capacity, are responsible for chemoresistance and relapse. Detection of these rare AML LCSs (approximately 1 per 1 × 10^6^ leukemic blasts) involved fluorescence-activated cell sorting (FACS) of the lymphoid-primed multipotent progenitors like CD34^+^CD38^−^CD90^−^CD45RA^+^ expressing cells and subsequent proof of identity by their ability to engraft in xenotransplantation assays [108]. The frequency of CD34^+^CD38^−^ LSCs among MRD leukemic blast cells were recently identified as a strong prognostic factor for poor overall patient survival [109,110]. Systematical protocols for flow cytometric detection and fractionation of MRD and LSCs are described elsewhere [57,111]. Using these procedures, Raffel et al. isolated and functionally validated CD34^+^CD38^−^ LSCs for TMT-based quantitative proteomics [87,88]. Comparison of LSCs vs. healthy hematopoietic stem and progenitor cells (HSPCs), as well as LSCs vs. leukemic blasts, demonstrated that processes such as RNA transport, DNA replication, and ribosome were enriched in leukemic blast cells, whereas metabolic pathways (e.g., citrate cycle and glycolysis/gluconeogenesis) were enriched in LSCs. Thus, these experiments emphasize the importance of combining cell sorting techniques with MS-based proteomics for the discovery of post-transcriptional regulatory changes in disease. 

Isolation and functional validation of LSCs is a time-consuming process, and therefore an unsuitable procedure for clinical implementation. One must also keep in mind that engraftment and in vivo propagation of human LSCs take place in a foreign microenvironment with mismatches in cytokines and other physiological factors. Unfortunately, the low number of LSCs makes it difficult to quantify FACS-isolated CD34^+^CD38^−^ LSCs fractions directly by LC-MS/MS. However, it was recently reported that more than 5000 proteins could be quantified from only 25,000 FACS-fractionated human HSPCs or common myeloid progenitors [112]. Therefore, it appears that the new development in the sensitivity and scan speed of the mass spectrometers in combination with improved high-throughput sample preparation and labeling methods (e.g., TMT) will make possible to quantify a small number of cells (<100) and even single cells by LC-MS/MS in the near future [113].

## 4. Implementation of Proteomic Workflows in Cancer Clinical Routines

### 4.1. Advanced Proteomic Workflows with Potential Clinical Use

Genetic markers can indicate increased risk or predisposition to develop a certain disease. However, these alone do not indicate the presence of disease. Therefore, phenotypic markers such as changes in protein expression or protein phosphorylation levels are required to do this. Thus, clinical proteomics research that focuses on discovering protein markers has been hailed as the “next step forward” in the early detection, diagnosis, and treatment of diseases. Using proteomics methods to investigate changes in protein expression or protein phosphorylation in cell cultures or tissue samples has led to the discovery of many potential biomarkers. However, biomarker validation requires studies involving hundreds of patients before they can be of use in clinical routines. This might explain why the number of validated disease biomarkers originally identified by MS-based discovery proteomics studies remains very low. As we have seen in AML proteomics studies, the preparation of proteome samples for MS-based proteomics studies is varied. Most of the sample preparation methodologies use detergents, chaotropes, and organic solvents at the cell lysis step. The impact of the traces of these chemicals on the mass spectrometer’s performance differs, being most detergents not compatible with downstream electrospray ionization (ESI) MS. Thus, more LC-MS/MS-friendly preparations such as those that employ formalin fixation and paraffin-embedding (FFPE) of tissue followed by cell lysis with TFE has achieved a proteomic depth of 5000–6500 proteins groups per single sample in a reproducible fashion [114]. This protocol characterized by a short sample preparation time (app. one day) assured a fast sample analysis that could be used for diagnosis and target identification routines.

MS technology keeps improving the coverage of the dynamic range of protein samples without committing the speed and sensitivity of the measurements. Trapped ion mobility spectrometry coupled with quadrupole TOF MS (timsTOF Pro) offers a unique dimension of characterization and separation in complex mixtures. The timsTOF Pro instrument which allows parallel ion sequencing by the Parallel Accumulation Serial Fragmentation (PASEF) method [115] and thereby multiplies the sequencing speed, represents MS devices of potential application in the clinic.

A few years ago, a promising automated, high reproducible, 3-h proteomics workflow for analyzing the plasma proteome from blood droplets was described [116]. As proteins in the circulatory system mirror an individual’s physiology and health state, a fast, reproducible, and sensitive protein profiling of plasma samples might be of enormous applicability in biomedicine. The workflow consisted of a sample preparation according to the in-StageTip (iST) procedure [117] (although automation for liquid handling platforms was also indicated and applied in a more recent plasma proteome profiling study [118]), peptide measurement in the Orbitrap instrument Q Exactive HF, and analysis by advanced label-free quantification logarithms in MaxQuant software [119]. More than 300 proteins were measured in a fast 20-min LC-MS/MS run with the help of a reference dataset of proteins from depleted and undepleted plasma. The intra-assay correlation was 0.98 and the coefficients of variation were smaller than 20% for the majority of quantified proteins. The workflow could be adjusted to other quantification strategies that employ mixtures of recombinant isotope-labeled protein fragments (SILAC-PrESTs) [120] or TMT. In a separate study, the proteome depth and quantitative reproducibility with the TMT method have been studied across three independent laboratories by Clinical Proteomic Tumor Analysis Consortium (CPTAC) investigators [121]. A maximum deviation across proteome replicates and laboratories less than 7% suggested the standardization of this sort of quantification method for clinical routines.

However, in-depth MS-based proteomics studies leading to the discovery of prognostic cancer biomarkers using tumor samples require larger amounts of proteins for sample processing, longer digestion procedures, optional peptide fractionation, and longer LC-MS/MS runs (in the case of working with unfractionated peptide samples). Thus, cancer/testis antigen 45 (CT45) was identified as an independent prognostic factor associated with a doubling of disease-free survival in advanced-stage high-grade serous ovarian cancer (HGSOC) when FFPE tumor samples were processed to yield 100 µg of protein for overnight digestion [122]. A 250 min LC-MS/MS runs of the label-free peptides quantified more than 9000 proteins from each chemotherapy-resistant and chemotherapy-sensitive patient tumor.

Protein phosphorylation plays a critical role in many biological processes of cancer development. Because of its low stoichiometry, an enrichment step is necessary to achieve a good depth of the phosphoproteome under study. The use of automated platforms to perform enrichment protocols with low sample amounts in a less labor-intensive, more sensitive, and less prone to variability fashion has increased in the past years. The AssayMAP Bravo platform, as an example, identified 6000 phosphopeptides from 50 µg of a label-free HeLa digest and more than 3500 peptides from 25 µg of a label-free rat hippocampal neuron digest with high reproducibility (average correlation of *R* = 0.87) using IMAC cartridges [123].

Taken together MS-based proteomics workflows for clinical use should be properly optimized regarding the amount of starting material, cell lysis and digestion procedures, fractionation or enrichment, and LC-MS/MS performance. Moreover, these methodology parameters will be sample dependent in many cases.

### 4.2. Bioinformatics Pipelines with Potential Clinical Use

Although the assessment and evaluation of current bioinformatics pipelines to analyze MS-generated proteomic data are beyond the scope of this review, herein we introduce systematic bioinformatics workflows that might be of use in clinical proteomics routines. To reduce the variability introduced by the use of different data analysis platforms for the analysis and comparison of CPTAC data (e.g., software packages, versions, search parameters, sequence databases, etc.), the Common Data Analysis Platform (CDAP) was developed [124]. The CDAP produces both peptide-spectrum-match (PSM) reports and gene-level reports. The pipeline processes raw MS data with ReAdW4Mascot2, MS-GF+, and NIST-ProMS programs to carried out peak-picking and quantitative data extraction, database searching, gene-based protein parsimony and false discovery rate (FDR)-based filtering. The pipeline also produces localization scores for phosphopeptide enrichment studies using the PhosphoRS program. Quantitative information is provided by the spectrum-level or gene-level precursor peak areas and by spectral counts for label-free or reporter ion log-ratios for 4plex iTRAQ labels.

Researchers with little or none experience with biocomputational analysis of MS-based proteomics data can benefit from free proteomics workflows offered by the de.NBI service center BioInfra.Prot. The workflow components include tools and methods for spectrum identification and protein interference, protein quantification, expression analysis as well as data standardization and data publication [125]. Tools and workflows are offered for different software environments (R, Java, KNIME, etc.). However, the preferred environment for users with little background in bioinformatics is KNIME (https://www.knime.com/), free software for interactive data analysis, which allows the integration of several methods through its modular pipelining concept. Other free services such as consulting or online training from fall 2020 are also provided [126]. Another project from the de.NBI, developed at the center for integrative bioinformatics, has provided OpenMS, an open-source software C++ library for LC-MS/MS data management and analysis [127]. This free software runs under Windows, macOS, and Linux. OpenMS processes proteomics data by combining a set of almost 200 ready-made tools from The OpenMS proteomics pipeline (TOPP). As these tools are available on the command line from within workflow engines such as KNIME and Galaxy, users are required to have some knowledge of informatics.

Users with more experience with proteomics data can use the windows-based trans-proteomic pipeline (TPP) which includes modules for the conversion of raw MS data, peptide identification, peptide validation, quantitation, protein assignment, and protein listing [128]. A tutorial is provided for users that do not have an advanced knowledge of proteomics, MS, or software engineering.

We and a large part of the proteomics community use the popular MaxQuant software [129,130] and the Perseus platform [131,132] to process LC-MS/MS proteomics raw data and to analyze (including statistical treatments) protein groups and phosphosites, respectively. Although both tools required some experience with MS-generated data and interpreting protein quantification and post-translational modification results, we believe that they could be easily implemented in clinical proteomics workflows. The developers are currently working on the workflow automation of Perseus [133]. Once this is done, it could be straightforward to run it together with MaxQuant in one single process.

## 5. Current Status on the Implementation of Proteomic Workflows in AML Clinical Routines

With regards to AML, as well as to other cancer diseases, the implementation of proteomic workflows in clinical routines is currently a long-term project. Up to date, most of the MS-based proteomics studies on AML patients have aimed the discovery of new diagnosis, prognosis, or drug response biomarkers. However, most of the identified biomarkers have not been further validated with external cohorts. The expression of some plasma membrane proteins in AML cells has been quantified by both MS-based and cytometry-based techniques (Table 2) but none of them has been further validated across human cohorts by MS-based proteomics.

Moreover, the AML research community lacks an online repository of MS-based proteomics experiments on AML patients that might allow comparative studies. As an example of these repositories and their applications, the CSF Proteome resource allows the comparison of the user’s data to its cerebrospinal fluid (CSF) data resource and inspect the proteomics data for other related neurological disorders [134,135]. 

Protocols for AML cell sample isolation (LSCs or blasts), storage (i.e., biobank curation, duration, and temperature), sample preparation, and analysis by LC-MS/MS vary among the different research groups although we have observed that in-solution digestion, high pH RP fractionation, and TMT labeling have frequently been used in the different proteomics workflows published in the last years (Table 2). Therefore, a SOP that includes workflows for preanalytical sample processing to assure clinical implementation of proteomic findings remains to be generated [136]. An example of the research community’s efforts towards the standardization of the preparation parameters for plasma proteome samples for MS analysis can be found at the HUPO plasma proteome project [137,138]. Alternatively, the use of standardized commercial solutions for proteome analysis (e.g., EVT Execute; https://www.evotec.com) might encourage agreement on the sample preparation for the MS analysis of AML patient cells in the clinic.

Taken together and contrary to other research areas as those that involved plasma or CSF samples, MS-based proteomic analysis of AML samples requires more intensive collaborations and consensual progress towards its future clinical implementation. AML cells in a sufficient amount for extensive studies should be easily available from either blood or bone marrow for almost all patients. However, the techniques for sampling, handling of samples, preparation of the cells, enrichment of leukemic cells, and procedures for freezing/storage/thawing (together with those to produce peptides suitable for LC-MS/MS analysis reviewed earlier) need to be carefully standardized as the basis for future scientific communication.

## 6. Other Considerations in Clinical AML Proteomics

As we have described in previous chapters, MS-based proteomics research is a very dynamic field characterized by continuous improvements of sample preparation protocols compatible with LC-MS/MS (including those that involve artificial intelligence-guided platforms for tissue handling) and the quick development of faster and more sensitive mass spectrometers.

The MS-based AML proteomics studies described in this review have all used the data-dependent acquisition (DDA) approach for MS analysis. The DDA mode uses knowledge obtained during the acquisition to decide what MS1 peptide precursors to subject for fragmentation (MS2) in the collision cell. In the past years, another acquisition mode in modern mass spectrometers called data-independent acquisition (DIA) has been employed in high-coverage proteome studies [139]. DIA, in contrast, performs predefined MS2 fragmentation and data collection regardless of sample content, which allows for more sensitive and accurate protein quantification compared to DDA [140]. DIA approaches typically rely on spectrum libraries that contain fragmentation and retention time information for peptide identification. Thus, it might be feasible to adopt the DIA technology into the SOPs of clinical samples.

With the potential applications of MS-based proteomics in the characterization of AML samples, this strategy should be considered as an important support of the current genomics and genetics studies that are typically performed in AML clinics. Although metabolomics needs extensive development before it can be implemented as a clinical tool, we can envision that the interaction of these MS-based -omics strategies might be crucial to understand the complexity of highly heterogeneous diseases such as AML and thus help clinicians to make efficient decisions.

## 7. Discussion and Conclusions

MS-based proteomics represents a powerful strategy with multiple applications in the clinic. Apart from the identification and quantification of a disease’s proteome and relevant protein modifications as phosphorylation in global biomarker discovery studies with DDA and DIA methodologies, validated and specific disease biomarkers can be quantified by MRM conducted on triple quadrupole instruments or parallel reaction monitoring (PRM) on Q-TOF and Orbitrap instruments, respectively [141]. MS combined with other techniques such as flow cytometry (i.e., mass cytometry [136]) gives the opportunity to analyze different AML subpopulations. Although proteome analysis of human plasma samples seems to become a routine test in the near future, MS-based proteomics on AML samples for clinical assays appears to require more consensus and investigations.

MS-based proteomics could be of use to find well-validated prognosis, relapse, drug response, and drug resistance biomarkers in the test sample. In addition, longitudinal check-ups by MS-proteomics could be also used to monitor the remission of AML patients. However, before we can use this technique for those purposes, the AML research community needs to identify disease markers by (i) extensive validation with external cohorts and (ii) establishing SOPs to perform such validation studies in the different research laboratories.

We have noticed that while there is a major interest in the development and optimization of robotic automated liquid handling workstations, mass spectrometers, and bioinformatic pipelines for automated analysis, more efforts are needed to assess overall sample quality and consensual SOPs for sample isolation, storage, and preparation for LC-MS/MS analysis. A recent work has shown the impact of erythrocyte, thrombocyte, and platelet contaminations in biomarker studies of plasma samples [142]. The authors have provided three panels of about 30 proteins each that can serve as a reference for each contamination type and as an online tool for quality control of plasma proteome samples (http://www.plasmaproteomeprofiling.org). Furthermore, the study described how several blood-taking types of equipment may result in statistically significant differences in protein levels confounding biomarker quantification. This study recommended 17 considerations to minimize systematic bias ranging from the immediate harvest of the plasma after centrifugation to discarding of the lowest layer of plasma to avoid recontamination with platelets. Thus, a sample quality assessment needs to be carried out for any other human sample, including the different AML samples described in this review.

In this review, we have tried to highlight the impressive progress and the multiple applicability of the MS technology in the clinic. We hope that upcoming AML and clinical proteomics conferences might promote closer research and clinical collaborations directing the consensual use of the MS strategy for clinical routines of AML patients.

## Figures and Tables

**Table 1:** The current basis for prognostication of acute myeloid leukemia (AML) patients.

Prognostic Factors	Reference(s)
Circulating blast cells at the time of diagnosis	[49]
Karyotype	[12]
Molecular genetics	[12]
Secondary AML	[50]
Metabolic status at the time of diagnosis	[51]
AML subclones at the time of diagnosis	[52,53,54]
No remaining blasts on light microscopy 14–17 days after start of induction chemotherapy	[55]
Complete hematological remission after first induction cycle	[49]
MRD after consolidation	[56,57]
Time of relapse: - After conventional therapy (i.e., duration of first complete remission)	[58,59]
- Time from ASCT until diagnosis of relapse	[60,61]

MRD: minimal residual disease; ASCT: allogeneic stem cell transplantation.

**Table 2:** Liquid chromatography-tandem mass spectrometry (LC-MS/MS)-based proteomics from recently (2017–2020) published studies of AML patient cohorts

MS-Based Quantification	Sample Preparation Components	Clinical and Pathological Findings/Identified Marker(s)	Cohort Size ^a^	Ref.
Label-free DDA	In-solution digestion, IP	Study on the protein tyrosine kinase-protein tyrosine phosphatase connections and effect on protein-phosphotyrosine signaling networks	12	[80]
Label-free DDA	FACS, in-solution digestion, high pH RP-fractionation	Altered expression of leukemia-enriched plasma membrane proteins on distinct AML subclones. Some of the proteins (e.g., IL3RA, TIM3, CD44, CD96, CD47, CD32, IL2RA, CD99, and CLEC12A) have been previously identified by other non-MS-based technologies	42	[81]
Label-free DDA	FASP	The constitutive release of mediators from primary AML differ from the intracellular protein levels	19	[82]
Label-free DDA, SRM	2D-DIGE, IMAC	Phosphoprotein profiles reveal blast differentiation and cytogenic risk stratification	62	[83]
Label-free DDA	FASP	Patient subsets with high constitutive cytokine release levels show high expression of proteins involved in intracellular signaling interacting with integrins, RAC1, and SYK. AML cells with low release show high expression of transcriptional regulators	16	[84]
Label-free DDA	FASP	Strong antiproliferative and proapoptotic effects of metabolic pathways inhibitors on AML patient cells	14	[48]
Super-SILAC DDA	FASP, mixed-mode fractionation, IMAC	Higher phosphorylation of transcription regulators decreased cytokine release and increased integrin expression on cells from AML patients with high constitutive activation of the PI3K-AKT-mTOR signaling pathway	20	[85]
Super-SILAC DDA	FASP, mixed-mode fractionation, IMAC	Transcription factors and proteins involved in mRNA splicing are highly expressed in AML cells with self-renewal capacity	15	[86]
Super-SILAC DDA	FASP, mixed-mode fractionation, IMAC	Enhanced phosphorylation and activation of the PI3K-AKT-mTOR pathway by insulin is coupled to reduced antiproliferative effects of metabolic inhibitors in AML patient subsets	14	[68]
Super-SILAC DDA	FASP, mixed-mode fractionation, IMAC	High expression of RNA processing proteins, low expression of V-ATPase proteins, and higher activity of CSK2 and CDKs could help predict chemoresistant AML relapse	41	[46]
Super-SILAC DDA	FASP, mixed-mode fractionation, IMAC	High expression of mitochondrial ribosomal subunit proteins, RNA processing proteins, DNA repair proteins, and high activity of CDKs at AML relapse	14 ^b^	[47]
TMT DDA	FACS, LSCs engraftment, in-solution digestion, high pH RP-fractionation	Characterization of the expression of cell adhesion molecules, proteins of the oxidative phosphorylation process, and spliceosome factors in LSCs	18 ^c^	[87]
TMT DDA	FACS, LSCs engraftment, in-solution digestion, high pH RP-fractionation	BCAT1 is enriched in LSCs and links BCAA metabolism to epigenomic and post-translational HIF1α regulation via αKG-dependent dioxygenase	18 ^c^	[88]
TMT DDA	FACS, membrane, and cytosol isolation, in-solution digestion	Protein modification and cytoskeleton reorganization proteins showed an altered abundance in the proteome of leukemic progenitor cells	5	[89]
iTRAQ DDA	Nuclear isolation, in-solution digestion, high-pH RP-fractionation	Over-expression of nuclear S100A4 in AML cells. Nuclear S100A4 is crucial for AML survival	15	[90]

^a^ Cohort size is expressed as the number of AML patients in the study cohort; ^b^ 14: the number here represents 14 paired samples (diagnosis/relapse) from seven patients; ^c^ 18: the number here represents 18 AML populations from 6 patients; TMT: Tandem mass tag; LSCs: Leukemia stem cells; DDA: Data-dependent acquisition; IP: Immunoprecipitation; FACS: Fluorescence-activated cell sorting; RP: Reversed-phase; IL3RA: Interleukin-3 receptor subunit alpha; TIM3: T-cell immunoglobulin mucin receptor 3; CD: Cluster of differentiation; IL2RA: Interleukin-2 receptor subunit alpha; CLEC12A: C-type lectin domain family 12 member A; FASP: Filter-aided sample preparation; SRM: Selective reaction monitoring; DIGE: Difference gel electrophoresis; IMAC: Immobilized metal affinity chromatography; RAC1: Ras-related C3 botulinum toxin substrate 1; SYK: Tyrosine-protein kinase syk; SILAC: Stable isotope labeling with amino acids in cell culture; Mixed-mode fractionation: polystyrenedivinylbenzene reversed phase sulfonate, SDB-RPS; CSK2: Casein kinase 2; CDK: Cyclin-dependent kinase; BCAT1: BCAA transaminase 1; BCAA: Branched-chain amino acid; HIF1α: Hypoxia-inducible factor 1-alpha; KG: Ketoglutarate; iTRAQ: Isobaric tag for relative and absolute quantitation.
